# Successful Milk Oral Immunotherapy Promotes Generation of Casein-Specific CD137^+^ FOXP3^+^ Regulatory T Cells Detectable in Peripheral Blood

**DOI:** 10.3389/fimmu.2021.705615

**Published:** 2021-11-23

**Authors:** Yi Zhang, Lei Li, Geneviève Genest, Wei Zhao, Dan Ke, Sabrina Bartolucci, Nils Pavey, Tho-Alfakar Al-Aubodah, Duncan Lejtenyi, Bahar Torabi, Moshe Ben-Shoshan, Bruce Mazer, Ciriaco A. Piccirillo

**Affiliations:** ^1^ Department of Otolaryngology-Head and Neck Surgery, Beijing Chaoyang Hospital, Capital Medical University, Beijing, China; ^2^ Department of Otolaryngology-Head and Neck Surgery, Xinhua Hospital, Shanghai Jiaotong University School of Medicine, Shanghai, China; ^3^ Department of Medicine, McGill University, Montréal, QC, Canada; ^4^ Program in Translational Research in Respiratory Diseases, Research Institute of the McGill University Health Centre, Montréal, QC, Canada; ^5^ Program in Infectious Diseases and Immunology in Global Health, Centre for Translational Biology, Research Institute of the McGill University Health Centre, Montréal, QC, Canada; ^6^ Department of Microbiology and Immunology, McGill University, Montréal, QC, Canada; ^7^ Centre of Excellence in Translational Immunology (CETI), Montréal, QC, Canada; ^8^ Division of Allergy Immunology and Clinical Dermatology, Montreal Children’s Hospital, McGill University, Montréal, QC, Canada

**Keywords:** allergy, milk immunotherapy, regulatory T cells, clinical trial, tolerance, desensitization

## Abstract

**Background:**

Oral immunotherapy (OIT) is an emerging treatment for cow’s milk protein (CMP) allergy in children. The mechanisms driving tolerance following OIT are not well understood. Regulatory T cells (T_REG_) cells are key inhibitors of allergic responses and promoters of allergen-specific tolerance. In an exploratory study, we sought to detect induction of allergen-specific T_REG_ in a cohort of subjects undergoing OIT.

**Methods:**

Pediatric patients with a history of allergic reaction to cow’s milk and a positive Skin Pick Test (SPT) and/or CMP-specific IgE >0.35 kU, as well as a positive oral challenge to CMP underwent OIT with escalating doses of milk and were followed for up to 6 months. At specific milestones during the dose escalation and maintenance phases, casein-specific CD4^+^ T cells were expanded from patient blood by culturing unfractionated PBMCs with casein *in vitro.* The CD4^+^ T cell phenotypes were quantified by flow cytometry.

**Results:**

Our culture system induced activated casein-specific FOXP3^+^Helios^+^ T_REG_ cells and FOXP3^-^ T_EFF_ cells, discriminated by expression of CD137 (4-1BB) and CD154 (CD40L) respectively. The frequency of casein-specific T_REG_ cells increased significantly with escalating doses of milk during OIT while casein-specific T_EFF_ cell frequencies remained constant. Moreover, expanded casein-specific T_REG_ cells expressed higher levels of FOXP3 compared to polyclonal T_REG_ cells, suggesting a more robust T_REG_ phenotype. The induction of casein-specific T_REG_ cells increased with successful CMP desensitization and correlated with increased frequencies of casein-specific Th1 cells among OIT subjects. The level of casein-specific T_REG_ cells negatively correlated with the time required to reach the maintenance phase of desensitization.

**Conclusions:**

Overall, effective CMP-OIT successfully promoted the expansion of casein-specific, functionally-stable FOXP3^+^ T_REG_ cells while mitigating Th2 responses in children receiving OIT. Our exploratory study proposes that an *in vitro* T_REG_ response to casein may correlate with the time to reach maintenance in CMP-OIT.

## Introduction

Cow’s milk allergy (CMA) affects close to 0.6% of children under 2-years of age (1, 2). Up to 80% of children are expected to outgrow CMA by adulthood (3), but persistent CMA is a major risk factor for anaphylaxis due to accidental milk ingestion in school age-children (4). Cow’s milk oral immunotherapy (CM-OIT) is emerging as an effective experimental approach to induce tolerance to milk protein, with up to 75% of patients successfully achieving desensitization (4–7).

However, there are still a number of patients who fail to achieve sustained unresponsiveness to CMP, lose their state of desensitization to CMP during the maintenance period or discontinue treatment despite the demonstrated clinical efficacy of CM-OIT (8). Furthermore, successful CM-OIT requires rigorous patient compliance, any deviation in protocol may prolong the length of time required to reach maintenance or increase the risk of developing an allergic reaction the scheduled CMP doses (9). Undoubtedly, individual differences in immunity can also contribute to the variable clinical outcomes observed in CM-OIT studies. Many efforts have been made to identify clinically relevant biomarkers that predict individual CM-OIT outcomes, none of which have been successful thus far (10, 11). Since the clinical response to CM-OIT is highly variable, developing biomarkers that successfully predict ability to achieve desensitization, time to reach maintenance or risk of developing adverse events during therapy would enable the individualization of CM-OIT and increase safety of the procedure.

Recently, investigators have focused on examining the upstream cellular mechanisms implicated in oral tolerance to food. Regulatory T cells (T_REG_), a class of CD4^+^ T cells expressing the transcription factor Forkhead box P3 (FOXP3), have been of particular interest given their key roles in induction and maintenance of peripheral tolerance to a plethora of self and non-self antigens (12). Allergen-specific T_REG_ cells can suppress both innate and adaptive arms of an allergic response, preventing mast cell activation, IL-4 production, Th2 cell development and IgE production by B cells (13).

T_REG_ cells can be readily measured in the peripheral blood and defects in their abundance and function have been implicated in the pathophysiology of food allergy (14). Indeed, mutations within the FOXP3 locus are associated with the development of severe food allergies due to a widespread loss of tolerance to innocuous antigens (15). Children with IgE-mediated food allergy have significantly lower FOXP3 expression compared to healthy controls (16, 17), and decreased frequencies in circulating T_REG_ cells after allergen exposure (18–20). In patients with peanut allergy, OIT increases both the abundance and suppressive function of T_REG_ cells as well as induces epigenetic changes such as hypomethylation of the FOXP3 locus required for maintenance of a stable suppressive T_REG_ cell phenotype (21). In children with milk allergy, those who tolerate baked milk have a higher frequency of peripheral blood casein-specific suppressive FOXP3^+^CD25^+^CD127^-^ T_REG_ cells compared to children who do not, and this correlates with a higher likelihood of achieving milk tolerance (14). Similarly, children who outgrow their milk allergy have higher levels of peripheral CD4^+^CD25^+^ T_REG_ cells and lower *in vitro* T-cell proliferative responses to ß-lactoglobulin than those who do not (22). However, while the frequencies of antigen-specific T_REG_ cells and their secreted cytokines (IL-10, TGFβ) increase during OIT (23), neither successfully predict OIT outcomes (10).

In addition to potential disease heterogeneity and methodological variations that may have contributed to failed prediction of OIT outcomes in these studies, lack of reliable human T_REG_ cell markers is a significant limitation. T_REG_ cells are a functionally heterogenous population (24, 25) and traditional markers like CD25, CD127 and FOXP3 do not adequately discriminate between T_REG_ from T_EFF_ cells particularly in settings of T cell activation like allergy (25, 26). Most commonly used T_REG_ markers are also inducible on effector T cells (T_EFF_) upon TCR-mediated activation, blurring the distinction between human T_REG_ and activated T_EFF_ cells, increasing the functional heterogeneity of the population and confounding the interpretation of results (25). Importantly, we have previously shown that expression of the transcription factor Helios alongside FOXP3, can reliably discriminate stably-suppressive T_REG_ cells from T_EFF_ cells in activated immune settings (25). Moreover, the differential expression of CD137 (4-1BB), a direct target of FOXP3, and CD154 (CD40 ligand) can further discriminate recently activated, functionally suppressive T_REG_ from activated T_EFF_ cells in human peripheral blood (27).

In this pilot CM-OIT clinical study, we performed in-depth, phenotypic characterization of CD4^+^ T cell subsets specific to casein, the major protein allergens in cow’s milk. We aimed to evaluate whether CM-OIT induced casein-specific, stably-suppressive FOXP3^+^Helios^+^ T_REG_ cells and whether this cellular response correlated with successful OIT. Here, we characterized casein-specific T_REG_ and T_EFF_ cell phenotypes, based on differential CD137 (4-1BB) and CD154 (CD40L) expression, respectively, at several time-points during CM-OIT in 7 pediatric patients that successfully achieved CMP desensitization. We hypothesized that successful CM-OIT would require the expansion of casein-specific CD137^+^ T_REG_ cells rather than the polyclonal expansion of total peripheral blood T_REG_. Here, we propose that peripheral casein-specific CD137^+^ T_REG_ responses during CM-OIT can be used to identify patients likely to achieve successful CMP desensitization and may correlate with CM-OIT time to reach maintenance.

## Material and Methods

### Human Subjects

Seven patients were recruited from a prospective randomized-controlled trial aiming to compare adverse events in patients undergoing CM-OIT to patients that continued to avoid CMP. This study was conducted at the Pediatric Allergy and Clinical Immunology Department of the Montreal Children’s Hospital (MCH) in Montreal, Quebec, Canada (4). Informed consent was obtained for every patient and the study was approved by the Research Ethics Board of the McGill University Health Center (PED-12-090).

**Figure 1:**
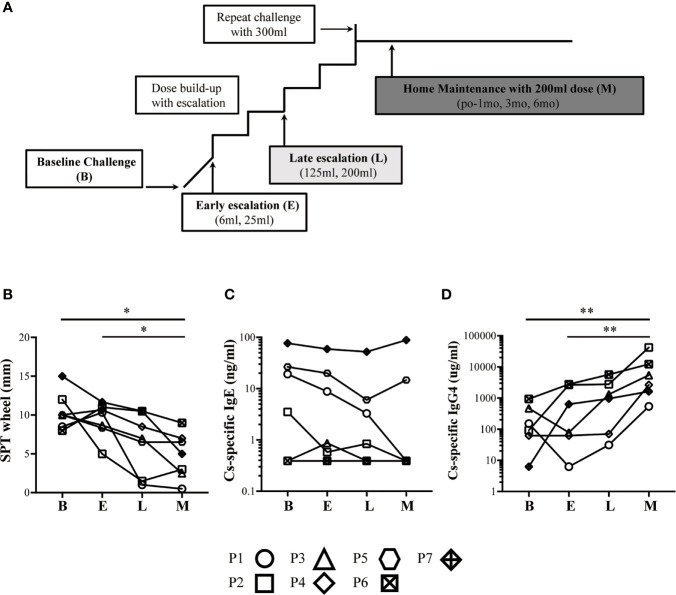
Successful OIT patients have increased levels of casein-specific IgG4 and whole milk SPT responses. **(A)** Typical approach to cow’s milk allergy immunotherapy. **(B)** SPT wheel size (mm) steadily decreased during CM-OIT in patients successfully achieving desensitization. **(C)** Casein-specific IgE (kU_A_/L) levels in successful OIT patients at baseline **(B)** did not decrease significantly during the early escalation phase E, late escalation phase L or months after reaching maintenance M. **(D)** Casein-specific IgG4 (kU_A_/L) steadily increased during CM-OIT in patients successfully achieving desensitization. Data is shown from 7 patients with each symbol representing a single patient. Casein-specific IgG4 levels were missing for P5. P-values were determined using a one-way ANOVA with a Dunn’s Multiple Comparison post-test (*p < 0.05, **p < 0.01).

Whole blood samples were obtained from 7 children who successfully completed CM-OIT (defined as successful challenge to 200 ml milk or 8000 mg milk protein) and from one healthy non-allergic control for comparison (26-year-old male), depicted in **Figure 4**. Briefly, for each study patient, IgE-mediated CMA was diagnosed by compatible clinical history and positive skin prick testing (SPT) with commercial CMP extract (≥3 mm over saline control) or positive serum casein-specific IgE levels (>0.35k U/L). Placebo-controlled single-blinded oral challenge to CM was used to confirm CMP allergy, and patients were assigned in a 1:1 ratio to either CM-OIT or CM avoidance for 1 year with crossover at the end of this period. The CM-OIT protocol started with rush desensitization and was followed by an early escalation phase (E; dose escalation from 6 ml to 25 ml of CM), a late escalation phase (L; dose escalation from 125 ml to 200 ml of CM) and a maintenance phase (M; maintained 200 ml of CM) (illustrated in **Figure 1A**). Blood samples were taken before OIT (baseline or B), during the E phase, the L phase, and 6 months after reaching the M phase (4).

### Peripheral Blood Mononuclear Cells and Lymphocyte Isolation

Whole blood samples were collected at B, E, L, M phase timepoints as well as from the healthy non-allergic control, as described above. PBMC were isolated from heparinized blood using Ficoll-based density gradient centrifugation. Isolated lymphocytes were labelled with CTV (Cell Trace Violet) or CFSE (carboxyfluorescein diacetate succinimidyl ester) and distributed into 96-well flat-bottom plates at a concentration of 5 × 10^5^ cells/well. Casein was dissolved in sodium hydroxide for 12 hours and adjusted to a pH of 7.3-7.4 with HCl before use. Lymphocytes were incubated with prepared casein protein (500μg/ml) or medium alone (RPMI 1640 supplemented with 10% Nu-serum) and cultured at 37°C in a 5% CO_2_ humidified incubator for 10 days, fresh media was replenished twice daily.

### IgE and IgG Detection

Milk/casein-specific serum immunoglobulins were measured by ELISA. The 96-well polystyrene plates were coated with casein or capture antibodies for IgE or IgG4. Casein was dissolved using 1M NaOH for 4 hours. The protein concentration was adjusted with coating buffer to 20 ug/ml. Capture antibodies were diluted 1:3000 with coating buffer (pH 9.6). The coated plates were incubated overnight at 4°C. Coated plates were washed twice with PBS-T containing PBS (pH 6.8) and 0.05% Tween 20. The plates were blocked with 1% bovine serum albumin (BSA) in PBS-T for 2 hours at room temperature (RT), washed, and 50 ul of milk OIT participant serum diluted in blocking buffer was added to the plates and incubated for 2 hours at RT. Each participant serum sample was added in duplicate.

Serial dilutions of known concentrations of IgE or IgG4 standard were added to wells coated with IgE or IgG4 capture antibodies. Blank wells, wells containing only blocking buffer, and well containing serum from non-milk allergic healthy volunteers were used as negative controls. Following four washes with PBS-T, the plates were incubated for one hour at RT with biotinylated goat anti-human IgE antibody diluted 1:3000 or biotinylated mouse anti-human IgG4 antibody diluted 1:250 in blocking buffer. The plates were then washed twice with PBS-T then incubated for one hour at RT with Streptavidin-HRP. After four washes with PBS-T, 50ul of tetramethylbenzidine (TMB) was added to each well then incubated for 15 minutes at RT. The reaction was stopped with 50ul of 1M phosphoric acid. The optical density was measured at 450nm with a reference wavelength of 570nm. Values were converted from ng/mL to kU/L by dividing by a factor of 2.4.

### Multi-Parametric Flow Cytometry

Lymphocytes were collected and stained with Viability dye (Fixable Viability Dye eFluor™ 780) and fluorescent monoclonal antibodies: anti-CD3-BV785 (clone OKT3), CD4-FITC (RPA-T4), CD8-PerCp-Cy5.5 (RPA-T8) and CD137-BV650 (4B4-1). Additional intracellular staining with anti-FOXP3-PE (206D), Helios-PE-Cy7 (22F6), CD154-APC (24–31) was performed after fixation/permeabilization of the cells using the Foxp3/Transcription Factor Staining Buffer Set (eBioscience). Detection of intracellular cytokines was performed by stimulating lymphocytes with Phorbol 12-myristate 13-acetate (PMA) (25 ng/ml) and ionomycin (1 μg/ml) (Sigma-Aldrich) in the presence of the Monensin-based Golgi inhibitor, Golgi Stop (BD Bioscience) for 3 h. Cells were stained using the same strategy as before, except CD4-AF700 (clone RPA-T4) was stained intracellularly following fixation/permeabilization. We evaluated cytokine production by staining with IL-4-PerCP-Cy5.5 (8D4-8) and IFN-γ-BV605 (B27) antibodies. Cells were acquired on a BD LSRFortessa X flow cytometer (BD Bioscience) and analyzed using FlowJo version 10 software (FlowJo, LLC).

### Statistical Analysis

A non-parametric one-way ANOVA followed by a Dunn’s Multiple Comparison post-test was used for longitudinal comparisons of parameters across more than two phases of the study (SPT wheal size, casein-specific sIgE and sIgG levels, changes in the proportions of peripheral T_REG_ subsets), while a Wilcoxon Signed Rank test was used for longitudinal comparisons across two phases only (frequencies of peripheral Th1 and Th2 cells). To determine correlations between CD137^+^ T_REG_ cells and cytokine-producing T_EFF_ cells or number of escalation days, we conducted a Pearson correlation. For comparisons of cell proportions or protein expression (MFI) between two or more T cell populations within a single phase of our study, a Wilcoxon Signed Rank was employed. Parametric unpaired student’s t-test or two-way ANOVA with Tukey’s post-testing were used to determine significance in *in vitro* experiments completed in triplicates from a single individual. A two-sided p-value of <0.05 was considered statistically significant. Statistical analyses were performed using Prism 7 Software (GraphPad, San Diego, CA).

## Results

### Successful OIT Patients Show Decreased Cow’s Milk SPT and Increased Casein-Specific IgG4 Responses

The details of the global trial design were recently published and is depicted in **Figure 1A** (4). Seven children from this cohort who successfully achieved CMP-OIT maintenance dosing were randomly selected for this study. Baseline demographics and clinical characteristics of all subjects are outlined in **Table 1**. The mean age was 12 years and 4/7 were female (57%). All patients reached the target maintenance dose of 200 ml with an average escalation period of 266 days (range: 168-504, IQR=98). The mean cow’s milk SPT was 10.5 mm (range: 8-15, IQR=1.75) at study entry and 4.79 mm (range: 0.5-9, IQR=4) after 6 months of CM-OIT maintenance, representing a significant decrease from baseline (p=0.03) (**Figure 1B**). Casein-specific sIgE were available in all 7 patients but sIgG4 levels were only available for 6/7 patients. No significant changes in casein-specific IgE levels were detected during the study period (p=0.15) (**Figure 1C**), whereas casein-specific IgG4 increased in all patients by the M phase (p=0.0071) (**Figure 1D**). There was no correlation between SPT size, casein-specific IgE or IgG4 levels and individual time required to reach maintenance (data not shown).

**Table 1:** Baseline patient characteristics.

	Age (years) /Gender	Cumulative dose (ml)	Systemic allergy	SPT (mm)				Casein-specific IgE (ng/ml)				Casein-specific IgG4 (μg/ml)				Escalation days
				B	E	L	M	B	E	L	M	B	E	L	M	
P1	7/M	0.1	Asthma	8.5	10.33	1	0.5	19.23	8.81	3.29	0.39	151.33	6.25	31.25	538.1	196
			Eczema													
			AR													
P2	15/F	14.4	Asthma	12	5	1.5	3	3.53	0.58	0.84	0.39	92.42	2672.38	2790.75	42219.36	252
P3	12/M	89.4	Asthma	10	8.67	7	2.5	0.39	0.87	0.39	0.39	468.62	78.69	1320.29	5416.46	238
P4	14/F	89.4	Asthma Eczema	10	10. 67	8.5	7	0.39	0.39	0.39	0.39	62.5	62.5	70.20	2661.97	182
P5	12/F	44.4	Asthma Eczema	10	8.33	6.5	6.5	26.7	19.96	6.03	14.75					168
P6	12/F	1.4	Asthma Eczema	8	11	10.5	9	0.39	0.39	0.39	0.39	940.28	2779.05	5705.03	12276.90	504
P7	14/M	0.1	Asthma Eczema	15	11.67	10.5	5	76.77	59.2	52.19	88.63	6.25	633.38	964.94	1639.09	322

B, Baseline; E, Escalation; L, Late L; M, Maintenance.

### Desensitization Is Associated With Casein-Specific T_EFF_ Cells With Altered Cytokine-Secreting Potentials

**Figure 2:**
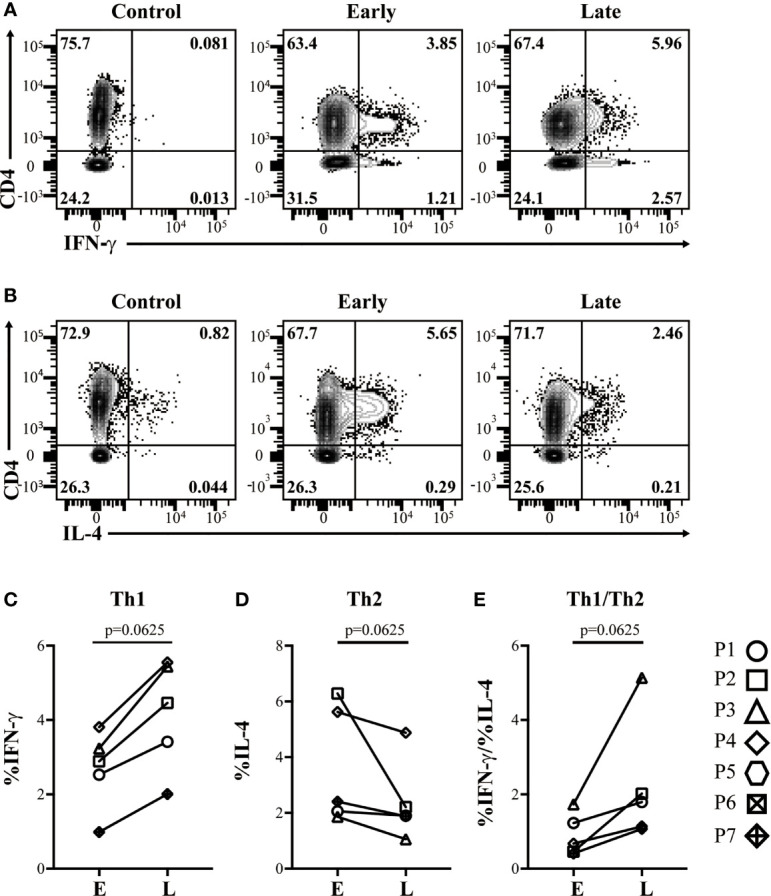
Successful desensitization is characterized by expansion of IFN-γ-producing, but not IL-4-producing T_EFF_ cells following *in vitro* restimulation with casein. Representative flow cytometry plots from controls lacking PMA stimulation, early phase and late phase identifying **(A)**, CD4^+^ IFN-γ^+^ T_EFF_ cells, and **(B)** CD4^+^ IL-4^+^ T_EFF_ cells emerging in patient PBMC after a 10 day culture in the presence of casein. **(C)** Proportions of CD4^+^ IFN-γ^+^ T_EFF_ cells increased with dose escalation. **(D)** Proportions of CD4^+^ IL-4^+^ T_EFF_ cells from culture with casein decreased with dose escalation. **(E)** Ratios of CD4^+^ IFN-γ^+^ T_EFF_ to CD4^+^ IL-4^+^ T_EFF_ from culture with casein increased with dose escalation. Data is shown from 5 patients. P-values were determined using a Wilcoxon Signed Rank non-parametric test.

PBMC from each study subject was cultured with casein or Tetanus Toxoid (TT) for 10 days before T cell profiles were evaluated by flow cytometry. CM-OIT dose escalation was associated with the increased expansion of IFN-γ-producing Th1 (CD4^+^Foxp3^-^) cells following *in vitro* casein challenge (**Figures 2A, C**, P=0.0625). In contrast, IL-4-producing Th2 cell expansion following casein challenge tended to decrease during CM-OIT dose escalation (**Figures 2B, D**, P=0.0625). Correspondingly, the ratio of Th1 to Th2 cells increased between E and L phases (**Figure 2E**, P=0.0625), albeit not significant. Analysis of Th1 and Th2 cells were only completed on 5 patients during E and L phases due to sample availability. Our data demonstrates a deviation in circulating Th2 responses towards Th1 immunity over the course of CM-OIT.

### Casein-Specific Expansion of Stably-Suppressive FOXP3^+^Helios^+^ T_REG_ Cells

**Figure 3:**
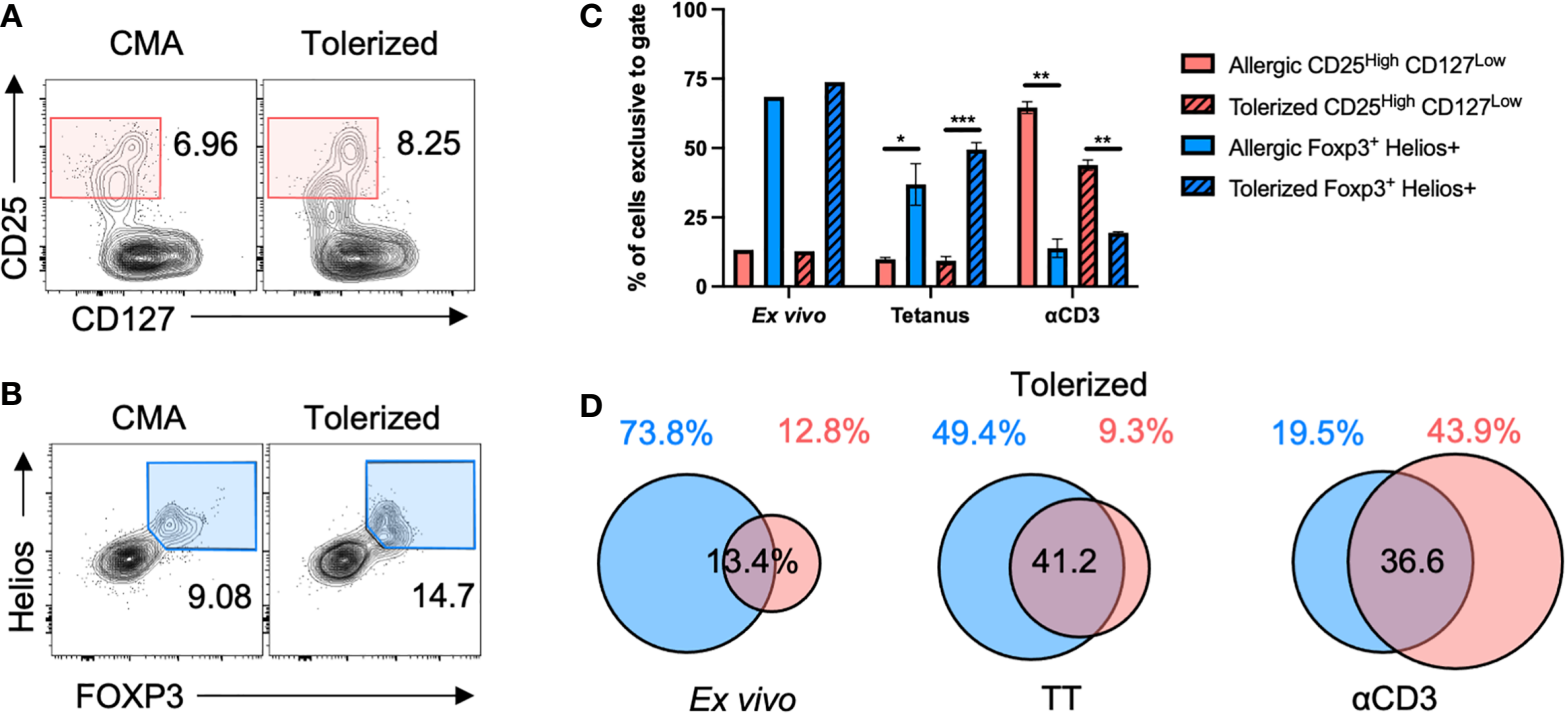
FOXP3^+^Helios^+^ is a stringent definition for T_REG_ cells. PBMC from a representative CMA patient before and after tolerization were stimulated with TT or αCD3 for 4 days before staining for T_REG_ cells in flow cytometry. **(A)** Sample flow cytometry plots showing CD25^High^CD127^Low^ T cells, and **(B)** FOXP3^+^Helios^+^ T_REG_ cells both pre-gated on CD4^+^ T cells. **(C, D)** The proportion of CD4^+^ cells captured by either CD25^High^CD127^Low^ gating or FOXP3^+^Helios^+^ gating that were exclusive to either CD25^High^CD127^Low^ or FOXP3^+^Helios^+^ gates were plotted in **(C)** with the degree of overlap between both populations shown in Euler-diagrams in **(D)** Cultures were completed in triplicates from a single patient’s PBMC (N=3). P-values were determined using a two-way ANOVA with a Tukey’s post-test (*p < 0.05, **p < 0.01, ***p < 0.001). Bars represent the mean ± s.d.

To evaluate a potential increase in immunoregulation with CM-OIT, we aimed to characterize T_REG_ cells both *ex vivo* and in our *in vitro* casein re-stimulation system. We compared the phenotypic definition of T_REG_ cells using traditional markers (CD25^High^ CD127^Low^) to T_REG_ cells defined by FOXP3 and Helios co-expression in a representative CMA patient before and after reaching maintenance dosing (**Figures 3A, B**). Indeed, we have previously shown that FOXP3^+^ Helios^+^ T_REG_ cells represent a stably suppressive population of T_REG_ in healthy individuals (24, 25). *Ex vivo* and following *in vitro* stimulation with TT (antigen-specific T cell activation), the CD25^High^CD127^Low^ gating excluded more than half of the FOXP3^+^Helios^+^ T_REG_ cells (**Figures 3C, D**). In contrast, after αCD3 stimulation (strong polyclonal T cell activation), the FOXP3^+^Helios^+^ gating was more stringent than CD25^High^CD127^Low^ gating with the latter definition also including FOXP3^–^ T_EFF_ cells and FOXP3^+^ Helios^–^ T_REG_ cells alongside FOXP3^+^Helios^+^ T_REG_ cells (**Figures 3C, D**). Thus, we decided to define T_REG_ cells as FOXP3^+^Helios^+^ in both CM-OIT and our *in vitro* culture systems.

**Figure 4:**
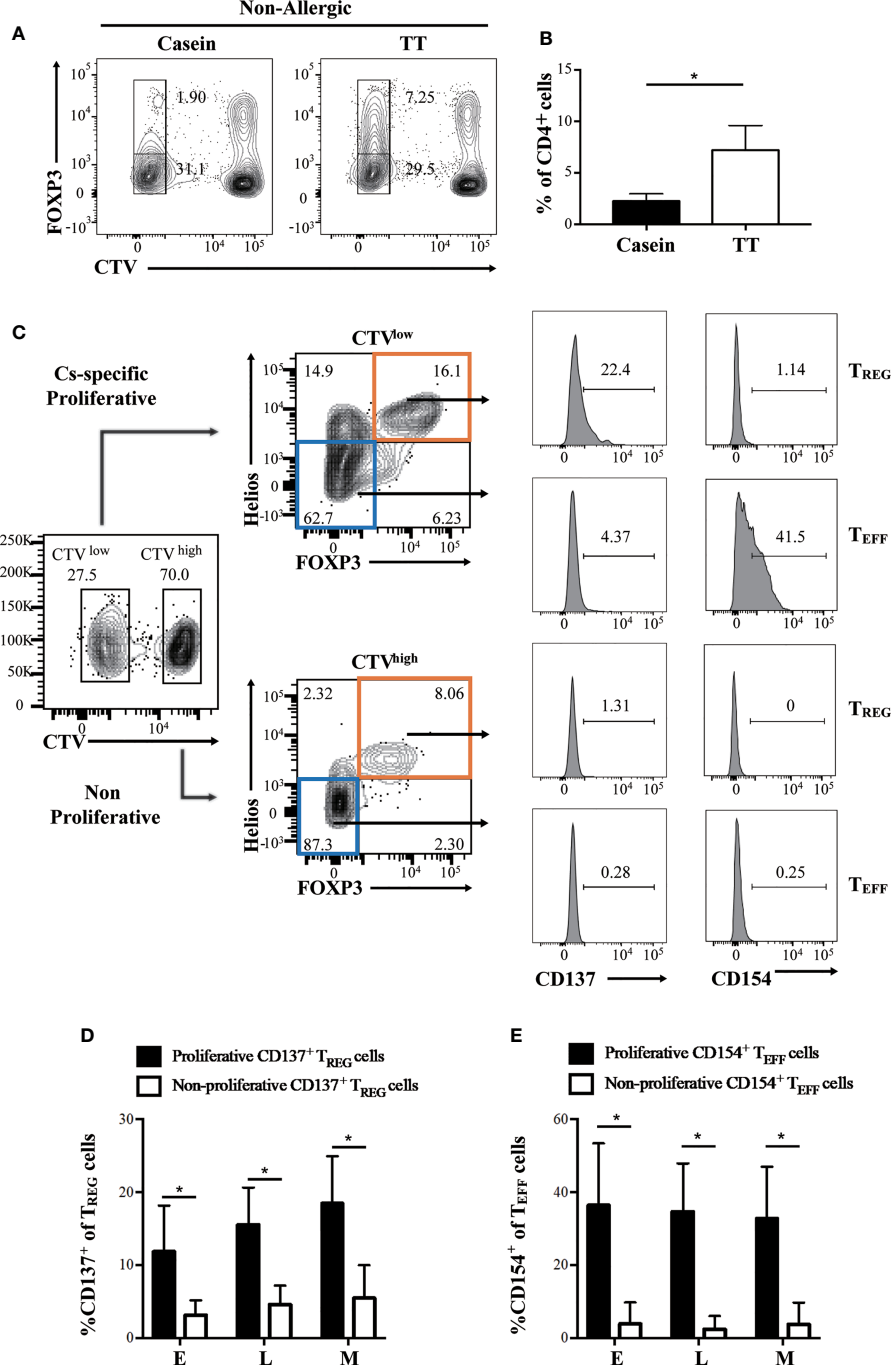
CD137 and CD154 differentially identify casein-specific T_REG_ and casein-specific T_EFF_ cells. Proliferation of CD4^+^ cells was assessed by flow cytometry-based CTV dilution analysis. **(A, B)** Healthy, non-allergic PBMC was cultured in the presence of casein or TT. **(A)** Representative flow cytometry plots of FOXP3^+^ T cells depicting CTV dilution in CD4^+^ T cells alongside **(B)**, the quantification (N=3). **(C–E)** Patient PBMC was cultured in the presence of casein for 10 days before evaluating expanded T cell responses by flow cytometry. **(C)** Flow cytometric gating strategy using a representative sample identifying proliferative (CTV^-^, top panel) and non-proliferative (CTV^+^, bottom panel) T_REG_ cells (FOXP3^+^Helios^+^) expressing CD137 and proportion of T_EFF_ (FOXP3^-^Helios^-^) expressing CD154 from a representative patient. **(D)** Expression of CD137 was significantly higher in proliferative FOXP3^+^Helios^+^ T_REG_ cells expanded in patient PBMC (N=3). **(E)** CD154 expression was significant higher in proliferative FOXP3^-^Helios^-^ T_EFF_ cells expanded in patient PBMC (N=3). The P-value in B was determined using unpaired t-test. P-values in **(C, E)** were determined using a Wilcoxon Signed Rank non-parametric test (*p < 0.05). Bars represent the mean ± s.d.

In healthy, non-allergic control conditions, casein stimulation elicited a weak FOXP3^+^Helios^+^ T_REG_ proliferative response compared to stimulation with TT (**Figures 4A, B**). However, in subjects with CMA, stimulation with casein elicited a robust proliferative response in FOXP3^+^Helios^+^ T_REG_ cells (**Figure 4C**), suggesting the presence of casein-specific T_REG_ cells circulating in these patients.

### Differential Expression of CD137 and CD154 Distinguish Casein-Specific T_REG_ Cells and T_EFF_ Cells, Respectively

**Figure 5:**
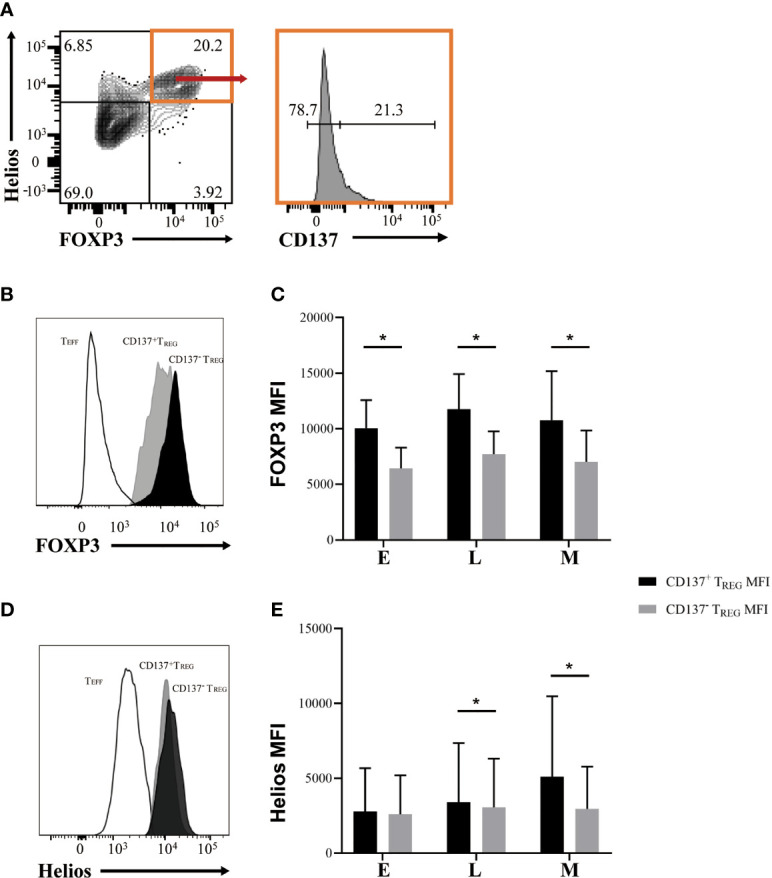
Casein-specific CD137^+^ T_REG_ cells express higher levels of FOXP3. **(A)** Representative flow cytometric plots identifying CD137^+^ and CD137^-^ T_REG_ (FOXP3^+^ Helios^+^) cells. **(B, C)** FOXP3 mean fluorescence intensity (MFI) is significantly higher in CD137^+^ T_REG_ cells than in CD137^-^ T_REG_ cells during all phases of CM-OIT (N=3). **(D, E)** Helios MFI is significantly higher in CD137^+^ T_REG_ cells than in CD137^-^ T_REG_ cells at L and M phases of CM-OIT (N=3). P-values were determined using a Wilcoxon Signed Rank non-parametric test (*P < 0.05). Bars represent the mean ± s.d.

Recently, it was suggested that CD137 and CD154 differential expression can identify antigen-specific T_REG_ and T_EFF_ cells in human PBMC, respectively (27, 28). Hence, to evaluate the presence of casein-specific T cells in our *in vitro* culture system, we utilized these markers. Proliferating T_REG_ cells were characterized by a significantly higher expression of CD137 than their non-proliferating counterparts (**Figures 4C, D**); similarly, proliferating T_EFF_ expressed higher levels of CD154 than non-proliferating T_EFF_ cells (**Figures 4C, E**). These results show that within all casein-specific T cells, CD137 expression is confined to proliferating T_REG_ cells whereas CD154 expression is confined to expanding T_EFF_ cells. CD137^+^ is a marker of proliferating casein-specific T_REG_ cells, whereas CD154^+^ is a marker of proliferating casein-specific T_EFF_ cells. We then evaluated the difference between CD137^+^ T_REG_ and CD137^-^ T_REG_ in terms of FOXP3 and Helios expression levels (**Figure 5**). While CD137^+^ T_REG_ cells expressed higher levels of FOXP3 at each timepoint (E, L, M) (**Figures 5B, C**), Helios was differentially expressed between CD137^+^ T_REG_ and CD137^-^ T_REG_ at the L and M phase (**Figures 5D, E**).

### Induction of Casein-Specific CD137^+^ T_REG_ Cells Correlates With Milk Sensitization, an Attenuated Th2 Response and Predicts the Length to Maintenance Phase

**Figure 6:**
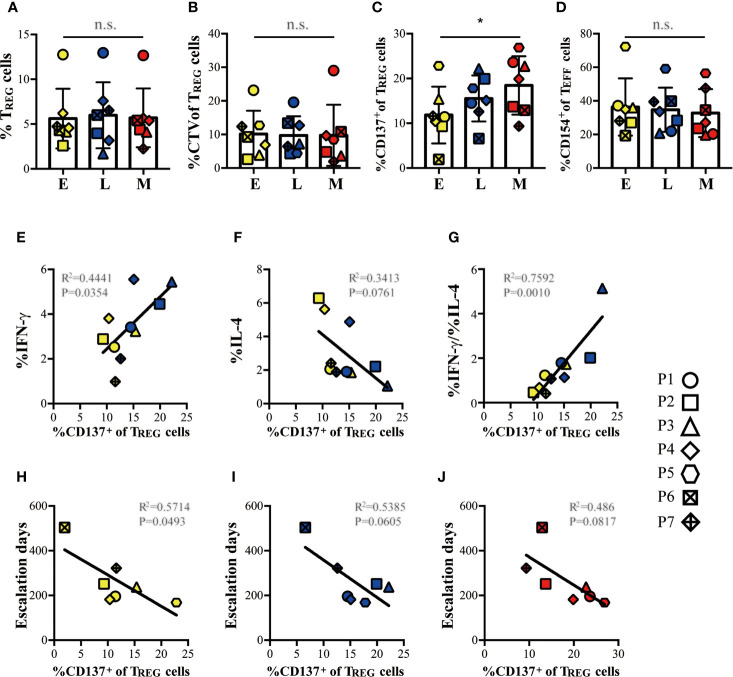
Induction of casein-specific T_REG_ cells correlated with tolerance, suppressed Th2 responses, and with escalation days to maintenance. **(A)** Proportion of Helios^+^FOXP3^+^ T_REG_ cells and **(B)**, proportion of proliferative (CTV^low^) Helios^+^FOXP3^+^ T_REG_ cells from total CD4^+^ T cells expanded in our *in vitro* culture system with casein do not change significantly during E, L and M phases of CM-OIT. **(C)** When differentiating T_REG_ based on CD137 expression, we observe that casein-specific CD137^+^ proliferative T_REG_ increase during Early, Late and Maintenance phase in successful CM-OIT patients. **(D)** There was no significant reduction in the proportions of CD154^+^ proliferative T_EFF_ cells during CM-OIT. **(E, G)** The induction of CD137^+^ proliferative T_REG_ correlated with an increase in the CD4^+^IFN-γ^+^ T_EFF_ cells from culture with casein and the ratio of CD4^+^IFN-γ^+^ T_EFF_ to CD4^+^IL-4^+^ T_EFF_ during Early and Late phase. **(F)** There was also a trend of correlation between CD137^+^ proliferative T_REG_ and CD4^+^ IL-4^+^ T_EFF_ cells from culture with casein, although there is a no significance. **(H)** There is a negative correlation between the proportions of CD137^+^ proliferative T_REG_ at **(E)** and escalation days to maintenance. **(I, J)** There was also a trend of correlation between the proportions of CD137^+^ proliferative T_REG_ at Late and Maintenance phase. and escalation days to maintenance, albeit no significance. Each symbol represents 1 subject. Of 7 patients, 5 patients from E and L phase are involved in analysis/figure **(E–G)**. Yellow symbols represent data at Early phase Blue symbols represent data at Late phase. Red symbols represent data at Maintenance phase. P-values in **(A–D)** were determined using a one-way ANOVA with Dunn’s multiple comparisons and in **(E–J)** with a Pearson correlation (*p < 0.05, n.s, not significant). Bars represent the mean ± s.d.

Since all patients successfully achieved the target CM-OIT maintenance dose, we sought to determine whether T_REG_ or T_EFF_ responses could be used as a marker of milk desensitization. Using the T_REG_ cell markers FOXP3 and Helios alone was insufficient to identify any differences in T_REG_ responses to *in vitro* casein challenge from PBMC isolated during E, L and M phases (**Figures 6A, B**). However, when stratifying T_REG_ cell responses based on CD137 expression, we observe that proliferating FOXP3^+^Helios^+^CD137^+^ T_REG_ cells steadily increased during successful CM-OIT (**Figure 6C**). The proportion of FOXP3^-^Helios^-^CD154^+^ T_EFF_ cells remained constant throughout the E, L and M phases (**Figure 6D**), suggesting that *in vitro* CD137^+^ T_REG_ cell induction rather than a reduction in antigen specific CD154^+^ T_EFF_ cell is associated with casein desensitization. Moreover, we found patients who reached maintenance phase under 36 weeks had highest frequency of FOXP3^+^Helios^+^CD137^+^ T_REG_ than patients with more than 36 weeks to maintenance phase at M (**Figure 6C**), suggesting higher frequency of FOXP3^+^Helios^+^CD137^+^ T_REG_ may be related to patients reaching M earlier. In early and late phases, the induction of FOXP3^+^Helios^+^CD137^+^ T_REG_ cells correlated with an increase in the frequency of T_EFF_ cells with a Th1 phenotype and Th1/Th2 ratio *in vitro* (**Figures 6E, G**). There was also a modest negative correlation between FOXP3^+^Helios^+^CD137^+^ T_REG_ and the frequency of T_EFF_ cells with a Th2 phenotype, albeit not significant (**Figure 6F**). Lastly, there is a negative correlation between the proportion of FOXP3^+^Helios^+^CD137^+^ T_REG_ and the number of escalation days required to reach maintenance at E (**Figure 6H**), this is also observed for L and M, albeit non-significant (**Figures 6I–J**). This suggests that FOXP3^+^Helios^+^CD137^+^ T_REG_ at E may correlate with individual time to reach maintenance.

## Discussion

Cow’s milk OIT is an effective treatment for inducing oral tolerance in milk-sensitized individuals. However, its clinical applicability is limited by the inability to predict the probability of achieving successful desensitization or sustained unresponsiveness. In this exploratory proof-of-concept study, we suggest that stably-suppressive, casein-specific CD137^+^ FOXP3^+^Helios^+^ T_REG_ may be a good candidate biomarker for identifying patients most likely to achieve successful CMP desensitization and be useful to predict time to reach maintenance in patients undergoing CM-OIT.

We characterized the immune parameters of 7 children with successful CM-OIT at several timepoints during treatment. We began by evaluating the standard published biomarkers, namely SPT to cow’s milk, casein-specific sIgE levels, casein-specific sIgG4 levels, as well as peripheral casein-specific Th1 and Th2 cells. As expected, casein-specific sIgE levels remained relatively stable during the study period, cow’s milk SPT size decreased and casein-specific sIgG4 levels increased with successful desensitization. Most patients maintained a positive SPT to cow’s milk and casein-specific sIgE levels in the maintenance phase, demonstrating an ongoing potential for reactivity to CMP despite clinical induction of desensitization.

Since allergen-specific T cell subsets are emerging as a potential prognostic indicator of OIT outcomes, we then examined at casein-specific T_EFF_ and T_REG_ subsets at each phase of our study. To identify casein-specific T cells, we labelled PBMC with either CTV or CFSE proliferation dyes to identify expanding (CTV^low^ or CFSE^low^) subsets upon exposure to casein. We observed an expansion of IFN-γ-producing T_EFF_ (Th1) cells from culture with casein, with a modest corresponding decrease in IL-4-producing T_EFF_ (Th2) cells between E and L phases, but this was not seen across the entire study period. This observation is in keeping with previous reports that CM-OIT induces a shift away from the predominant Th2 response to milk protein early during the desensitization process (3). Mechanisms of tolerance likely differ between dose escalation and maintenance phase which may explain why Th1 prominence only increased significantly during dose escalation in our study. Although T_EFF_ subsets may change during OIT, predictive thresholds, appropriate timing of sampling and robust correlations with clinical phenotypes are lacking, and further studies are required to validate their clinical usefulness (10). Of note, we did not find any correlation between T_EFF_ subtypes and the time to reach maintenance.

Induction of allergen-specific T_REG_ cells has classically been shown to be a later effect of OIT, and product of local differentiation of conventional T cells into allergen-specific T_REG_ cells following allergen exposure. These induced T_REG_ cells (iT_REG_) are less stable than their thymic-derived natural T_REG_ (tT_REG_) counterparts and have the potential to lose their suppressive phenotype under specific inflammatory contexts (29). Although the mechanisms of OIT mediating allergen tolerance have not been completely elucidated, stable T_REG_ induction seems to be central for the achievement and maintenance of CMP desensitization and loss of suppressive function or possible conversion of these cells to a Th2 cell phenotype could be associated with OIT failure (30). Previous studies have routinely evaluated T_REG_ in the clinic to predict OIT responses, but have been limited by the availability and choice of relevant surface markers to identify functional T_REG_ phenotypes (10). While both iT_REG_ and tT_REG_ cell subsets may be engaged in milk OIT, our results indicate that the emerging casein-specific T_REG_ cells express Helios, a transcription factor more frequently associated with T_REG_ cells of thymic origin (tT_REG_). Recently, however, Helios expression has also been shown to reflect T_REG_ stability and suppressive function, rather than mere T_REG_ lineage, as Helios acts to maintain the chromatin structure required for the induction and maintenance of the T_REG_ developmental program (31). Therefore, we interpret enhanced Helios expression as a marker of functionally suppressive T_REG_.

CD4^+^ T_REG_ cells have classically been defined by their expression of intracellular FOXP3, high cell surface expression of CD25 and low surface expression of CD127. However, CD25 and CD127 can be transiently modulated on CD4^+^ T_EFF_ cells upon immune activation and FOXP3 can be transiently expressed in T_EFF_ cells upon T cell receptor (TCR) ligation (32, 33). Furthermore, although FOXP3 reliably identifies T_REG_ in their resting, non-activated state, not all CD25^+^CD127^low^FOXP3^+^ T_REG_ clones are functionally suppressive (24). Thus, traditional markers of T_REG_ cells are not sufficient to identify functional and dysfunctional T_REG_ phenotypes.

Differential expression of a transcription factor of the Ikaros family, Helios, has been shown to reliably distinguish suppressive Helios^+^FOXP3^+^T_REG_ from non-suppressive Helios^-^FoxP3^+^ T_REG_ clones (25). However, CTV^low^CD4^+^FOXP3^+^Helios^+^ T_REG_ did not vary significantly during early, late and maintenance phases of CM-OIT in our study indicating that Helios may not be sufficient to identify allergen-specific T_REG_. Next, we sought to evaluate CD137 (4-1BB), a T_REG_ co-stimulatory receptor and a direct target of FOXP3 which has lately been identified as a robust marker of recently activated, antigen-specific, functionally suppressive iT_REG_ (27). Since effective T_REG_ suppression is antigen-specific, we hypothesized that successful CM-OIT would correlate with the expansion of casein-specific FOXP3^+^Helios^+^CD137^+^ T_REG_ cells (CD137^+^ T_REG_) rather than polyclonal T_REG_ activation or decrease in allergen-specific T_EFF_. In keeping with this hypothesis, we did observe that proliferating CD137^+^ T_REG_ significantly increase during early, late and maintenance phases of CM-OIT. Moreover, we found that the induction of CD137^+^ T_REG_ correlated with an increase in the frequency of T_EFF_ cells with a Th1 phenotype and a modest Th1/Th2 ratio suggesting that CD137^+^ T_REG_ suppress Th2 immune responses in CM-OIT. The negative correlation between frequencies of CD137^+^ T_REG_ cells and number of escalation days, and the finding that individuals with higher frequencies of CD137^+^ T_REG_ cells during the M phase needed less time to reach maintenance suggests that CD137^+^ T_REG_ may be useful for predicting time to reach maintenance during CM-OIT. To ensure that casein tolerance was possibly driven by CD137^+^ T_REG_ induction rather than a decrease in antigen-specific T_EFF_ cells, we compared proliferative T_EFF_ responses at each CM-OIT timepoint. Using CD154 as a marker of recently activated, antigen-specific T_EFF_ cells (27, 28), we found no significant difference in terms of proliferating CD4^+^FOXP3^-^Helios^-^CD154^+^ T_EFF_ cells (CD154 T_EFF_) throughout the study period.

Since a higher level of FOXP3 and Helios expression has been associated with increased suppressive potency and stability of the T_REG_ phenotype (25), we sought to determine differential expression of these two markers on CD137^+^ and CD137^-^ T_REG_ cells. Indeed, casein-specific CD137^+^ T_REG_ cells exhibited a higher level of FOXP3 expression than their CD137^-^ counterparts at each timepoint, whereas Helios was only differentially expressed between CD137^+^ T_REG_ and CD137^-^ T_REG_ at the M phase. These observations suggest that the circulating casein-specific CD137^+^ T_REG_ cells acquire a stable and more suppressive phenotype throughout CM-OIT, and that Helios expression, thus far not described in the OIT literature, may be utilized as a marker of successful OIT.

In summary, we have performed an exploratory CM-OIT study and identified a potential clinically useful biomarker to identify patients most likely to achieve successful CMP tolerance and sustained unresponsiveness during CM-OIT. This remains a pilot study and our conclusions will be validated in larger cohorts of patients which will include additional age appropriate non-allergic controls and patients having failed CM-OIT. The clinical utility of CD137^+^ T_REG_ quantification during CM-OIT merits further investigation and validation in larger cohorts.

## Data Availability Statement

The original contributions presented in the study are included in the article/supplementary material. Further inquiries can be directed to the corresponding author.

## Ethics Statement

The studies involving human participants were reviewed and approved by IRB of the McGill University Health Centre. Written informed consent to participate in this study was provided by the participants’ legal guardian/next of kin.

## Author Contributions

YZ, LL, GG, DK, SB, NP, DL, T-AA-A, and BT: sample processing, experimental design, assay development and execution, data analysis/reporting, and/or figure/manuscript preparation MB, BM, and CP: trial design, experimental design, data analysis and reporting, figure preparation, and manuscript preparation. All authors contributed to the article and approved the submitted version.

## Conflict of Interest

The authors declare that the research was conducted in the absence of any commercial or financial relationships that could be construed as a potential conflict of interest.

The reviewer BL declared a shared affiliation with one of the authors, LL, to the handling editor at time of review.

## Publisher’s Note

All claims expressed in this article are solely those of the authors and do not necessarily represent those of their affiliated organizations, or those of the publisher, the editors and the reviewers. Any product that may be evaluated in this article, or claim that may be made by its manufacturer, is not guaranteed or endorsed by the publisher.

## References

[1] Mousan G Kamat D. Cow’s Milk Protein Allergy. Clin Pediatr (Phila) (2016) 55(11):1054–63. doi: 10.1177/0009922816664512 27582492

[2] Soller L Ben-Shoshan M Harrington DW Knoll M Fragapane J Joseph L. Prevalence and Predictors of Food Allergy in Canada: A Focus on Vulnerable Populations. J Allergy Clin Immunol Pract (2015) 3(1):42–9. doi: 10.1016/j.jaip.2014.06.009 25577617

[3] Wood RA. Oral Immunotherapy for Food Allergy. J Investig Allergol Clin Immunol (2017) 27(3):151–9. doi: 10.18176/jiaci.0143 28102823

[4] De Schryver S Mazer B Clarke AE St Pierre Y Lejtenyi D Langlois A. Adverse Events in Oral Immunotherapy for the Desensitization of Cow’s Milk Allergy in Children: A Randomized Controlled Trial. J Allergy Clin Immunol Pract (2019) 7(6):1912–9. doi: 10.1016/j.jaip.2019.02.007 30776522

[5] Scurlock AM. Oral and Sublingual Immunotherapy for Treatment of IgE-Mediated Food Allergy. Clin Rev Allergy Immunol (2018) 55(2):139–52. doi: 10.1007/s12016-018-8677-0 29656306

[6] Keet CA Frischmeyer-Guerrerio PA Thyagarajan A Schroeder JT Hamilton RG Boden S. The Safety and Efficacy of Sublingual and Oral Immunotherapy for Milk Allergy. J Allergy Clin Immunol (2012) 129(2):448–55, 55 e1-5. doi: 10.1016/j.jaci.2011.10.023 22130425 PMC3437605

[7] Martorell A Alonso E Echeverria L Escudero C Garcia-Rodriguez R Blasco C. Oral Immunotherapy for Food Allergy: A Spanish Guideline. Immunotherapy Egg and Milk Spanish Guide (ITEMS Guide). Part I: Cow Milk and Egg Oral Immunotherapy: Introduction, Methodology, Rationale, Current State, Indications, Contraindications, and Oral Immunotherapy Build-Up Phase. J Investig Allergol Clin Immunol (2017) 27(4):225–37. doi: 10.18176/jiaci.0177 28731411

[8] Manabe T Sato S Yanagida N Hayashi N Nishino M Takahashi K. Long-Term Outcomes After Sustained Unresponsiveness in Patients Who Underwent Oral Immunotherapy for Egg, Cow’s Milk, or Wheat Allergy. Allergol Int (2019) 68(4):527–8. doi: 10.1016/j.alit.2019.02.012 30930020

[9] Nachshon L Goldberg MR Katz Y Levy MB Elizur A. Long-Term Outcome of Peanut Oral Immunotherapy-Real-Life Experience. Pediatr Allergy Immunol (2018) 29(5):519–26. doi: 10.1111/pai.12914 29698554

[10] Hardy LC Smeekens JM Kulis MD. Biomarkers in Food Allergy Immunotherapy. Curr Allergy Asthma Rep (2019) 19(12):61. doi: 10.1007/s11882-019-0894-y 31797153

[11] Eapen AA Lavery WJ Siddiqui JS Lierl MB. Oral Immunotherapy for Multiple Foods in a Pediatric Allergy Clinic Setting. Ann Allergy Asthma Immunol (2019) 123(6):573–81 e3. doi: 10.1016/j.anai.2019.08.463 31494236 PMC8215522

[12] Alvarez F Al-Aubodah TA Yang YH Piccirillo CA. Mechanisms of TREG Cell Adaptation to Inflammation. J Leukoc Biol (2020) 108(2):559–71. doi: 10.1002/JLB.1MR0120-196R 10.1002/JLB.1MR0120-196R 32202345

[13] Noval Rivas M Chatila TA. Regulatory T Cells in Allergic Diseases. J Allergy Clin Immunol (2016) 138(3):639–52. doi: 10.1016/j.jaci.2016.06.003 PMC5023156 27596705

[14] Shreffler WG Wanich N Moloney M Nowak-Wegrzyn A Sampson HA. Association of Allergen-Specific Regulatory T Cells With the Onset of Clinical Tolerance to Milk Protein. J Allergy Clin Immunol (2009) 123(1):43–52.e7. doi: 10.1016/j.jaci.2008.09.051 19130927

[15] Tordesillas L Berin MC. Mechanisms of Oral Tolerance. Clin Rev Allergy Immunol (2018) 55(2):107–17. doi: 10.1007/s12016-018-8680-5 PMC6110983 29488131

[16] Krogulska A Borowiec M Polakowska E Dynowski J Mlynarski W Wasowska-Krolikowska K. FOXP3, IL-10, and TGF-Beta Genes Expression in Children With IgE-Dependent Food Allergy. J Clin Immunol (2011) 31(2):205–15. doi: 10.1007/s10875-010-9487-1 PMC3105233 21107665

[17] Krogulska A Polakowska E Wasowska-Krolikowska K Malachowska B Mlynarski W Borowiec M. Decreased FOXP3 mRNA Expression in Children With Atopic Asthma and IgE-Mediated Food Allergy. Ann Allergy Asthma Immunol (2015) 115(5):415–21. doi: 10.1016/j.anai.2015.08.015 26409874

[18] Dang TD Allen KJ D JM Koplin JJ Licciardi PV Tang ML. Food-Allergic Infants Have Impaired Regulatory T-Cell Responses Following *In Vivo* Allergen Exposure. Pediatr Allergy Immunol (2016) 27(1):35–43. doi: 10.1111/pai.12498 26456457

[19] Masthoff LJN Pasmans S van Doorn H den Hartog Jager CF Geneugelijk K Knol EF. Major Hazelnut and Peanut Allergens are Potent in Basophil Activation and Cross-React at T-Cell Level. Allergy (2018) 73(10):2080–2. doi: 10.1111/all.13498 29885257

[20] Heeringa JJ Rijvers L Arends NJ Driessen GJ Pasmans SG van Dongen JJM. IgE-Expressing Memory B Cells and Plasmablasts are Increased in Blood of Children With Asthma, Food Allergy, and Atopic Dermatitis. Allergy (2018) 73(6):1331–6. doi: 10.1111/all.13421 29380876

[21] Syed A Garcia MA Lyu SC Bucayu R Kohli A Ishida S. Peanut Oral Immunotherapy Results in Increased Antigen-Induced Regulatory T-Cell Function and Hypomethylation of Forkhead Box Protein 3 (FOXP3). J Allergy Clin Immunol (2014) 133(2):500–10. doi: 10.1016/j.jaci.2013.12.1037 PMC4121175 24636474

[22] Karlsson MR Rugtveit J Brandtzaeg P. Allergen-Responsive CD4+CD25+ Regulatory T Cells in Children Who Have Outgrown Cow’s Milk Allergy. J Exp Med (2004) 199(12):1679–88. doi: 10.1084/jem.20032121 PMC2212808 15197226

[23] Satitsuksanoa P Jansen K Globinska A van de Veen W Akdis M. Regulatory Immune Mechanisms in Tolerance to Food Allergy. Front Immunol (2018) 9:2939. doi: 10.3389/fimmu.2018.02939 30619299 PMC6299021

[24] d’Hennezel E Yurchenko E Sgouroudis E Hay V Piccirillo CA. Single-Cell Analysis of the Human T Regulatory Population Uncovers Functional Heterogeneity and Instability Within FOXP3+ Cells. J Immunol (2011) 186(12):6788–97. doi: 10.4049/jimmunol.1100269 21576508

[25] Bin Dhuban K d’Hennezel E Nashi E Bar-Or A Rieder S Shevach EM. Coexpression of TIGIT and FCRL3 Identifies Helios+ Human Memory Regulatory T Cells. J Immunol (2015) 194(8):3687–96. doi: 10.4049/jimmunol.1401803 PMC4610024 25762785

[26] Attias M Al-Aubodah T Piccirillo CA. Mechanisms of Human FoxP3(+) Treg Cell Development and Function in Health and Disease. Clin Exp Immunol (2019) 197(1):36–51. doi: 10.1111/cei.13290 30864147 PMC6591147

[27] Weissler KA Rasooly M DiMaggio T Bolan H Cantave D Martino D. Identification and Analysis of Peanut-Specific Effector T and Regulatory T Cells in Children Allergic and Tolerant to Peanut. J Allergy Clin Immunol (2018) 141(5):1699–710 e7. doi: 10.1016/j.jaci.2018.01.035 29454004 PMC5938104

[28] Bacher P Heinrich F Stervbo U Nienen M Vahldieck M Iwert C. Regulatory T Cell Specificity Directs Tolerance Versus Allergy Against Aeroantigens in Humans. Cell (2016) 167(4):1067–78.e16. doi: 10.1016/j.cell.2016.09.050 27773482

[29] Noval Rivas M Burton OT Wise P Charbonnier LM Georgiev P Oettgen HC. Regulatory T Cell Reprogramming Toward a Th2-Cell-Like Lineage Impairs Oral Tolerance and Promotes Food Allergy. Immunity (2015) 42(3):512–23. doi: 10.1016/j.immuni.2015.02.004 PMC4366316 25769611

[30] Abdel-Gadir A Schneider L Casini A Charbonnier LM Little SV Harrington T. Oral Immunotherapy With Omalizumab Reverses the Th2 Cell-Like Programme of Regulatory T Cells and Restores Their Function. Clin Exp Allergy (2018) 48(7):825–36. doi: 10.1111/cea.13161 PMC6021220 29700872

[31] Thornton AM Shevach EM. Helios: Still Behind the Clouds. Immunology (2019) 158(3):161–70. doi: 10.1111/imm.13115 PMC6797934 31517385

[32] Gavin MA Torgerson TR Houston E DeRoos P Ho WY Stray-Pedersen A. Single-Cell Analysis of Normal and FOXP3-Mutant Human T Cells: FOXP3 Expression Without Regulatory T Cell Development. Proc Natl Acad Sci USA (2006) 103(17):6659–64. doi: 10.1073/pnas.0509484103 PMC1458937 16617117

[33] Allan SE Crome SQ Crellin NK Passerini L Steiner TS Bacchetta R. Activation-Induced FOXP3 in Human T Effector Cells Does Not Suppress Proliferation or Cytokine Production. Int Immunol (2007) 19(4):345–54. doi: 10.1093/intimm/dxm014 17329235

